# P-1569. Healthcare Disparities Between Racial, Ethnic, and Socioeconomic Backgrounds in Urinary Tract Infection Patients

**DOI:** 10.1093/ofid/ofae631.1736

**Published:** 2025-01-29

**Authors:** Ryan J Moazamian, Ashley Amundson, Henry S Fraimow, Carlo Foppiano Palacios

**Affiliations:** CMSRU, Sewell, New Jersey; CMSRU, Sewell, New Jersey; Cooper University Hospital and Cooper Medical School of Rowan University, Camden, NJ; Cooper University Health Care, Camden, New Jersey

## Abstract

**Background:**

Numerous studies have highlighted persistent health disparities: chronic disease rates, access to care, and outcomes across diverse races, ethnicities, and socioeconomic backgrounds. To address underlying healthcare disparities, we explored differences in outcomes of patients with urinary tract infections (UTIs).

**Table 1:**
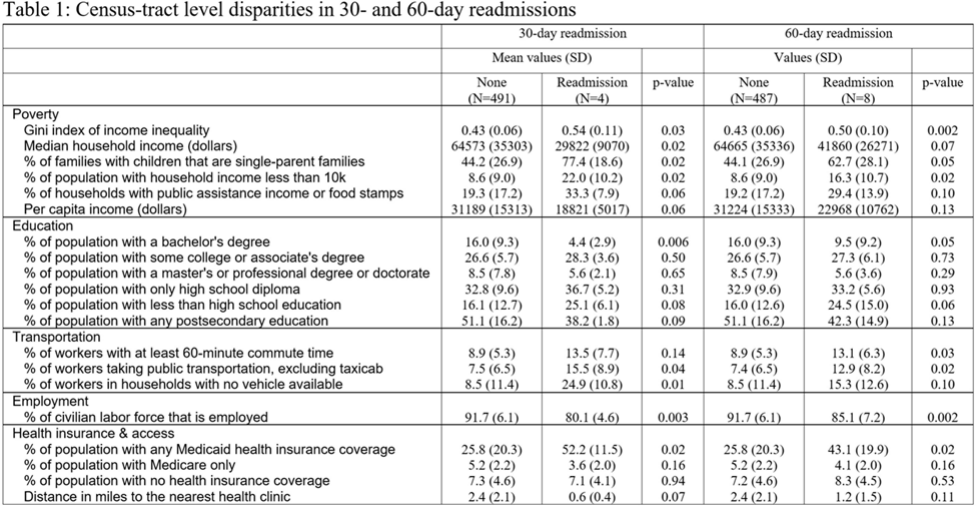
Results of Statistical Analysis

**Methods:**

Patients treated at Cooper University Hospital for UTI between 2019 and 2021 were identified using ICD codes. Inclusion criteria was age over 18, inpatient admissions longer than 48 hours, and diagnosis of UTI. Patient demographics, culture results, treatment, discharge outcomes, and outcomes at 30 and 90 days. Additionally, we collected individual indicators of socioeconomic status, including census tract data associated with American Community Survey, Area Deprivation Index (ADI: ranked on a scale 1 to 10, with 10 being most disadvantaged), and Social Vulnerability Index (SVI: ranked on a scale of 0 to 1, with 1 being most vulnerable). Descriptive statistics, Chi-square, and Kruskal-Wallis tests were performed.

**Results:**

495 patients with UTI were analyzed: mean age was 67±19 years, 54% were White, and 20% were Latino. Social Mean state ADI was 8.7±2.0, and mean CDC SVI was 0.63±0.24. Social determinants of health were common among patients with UTI: 60% did not have independent level of function, 19% lived alone, 18% had prior mental health history, 14% did not speak English, 8% had history of substance abuse, 2% had housing insecurity. Statistical analysis showed no meaningful difference in patient outcomes for hospital mortality or mortality at 30- or 60-days post discharge. Readmission at 30 or 60 days was associated with census tract median household income, household income less than 10 thousand dollars, per capita income, education with bachelor’s degree, taking public transportation, not having a car, unemployment, and Medicaid coverage (Table 1).

**Conclusion:**

We found no differences in mortality outcomes among patients with UTI across various socioeconomic parameters within our institution. However, hospital readmissions were more likely among patients who lived in census tracts with increased hardships. Future studies should evaluate possible interventions to decrease readmission disparities.

**Disclosures:**

**All Authors**: No reported disclosures

